# Advancing Our Understandings of Healthcare Team Dynamics From the Simulation Room to the Operating Room: A Neurodynamic Perspective

**DOI:** 10.3389/fpsyg.2019.01660

**Published:** 2019-08-12

**Authors:** Ronald Stevens, Trysha Galloway, Ann Willemsen-Dunlap

**Affiliations:** ^1^UCLA School of Medicine, Brain Research Institute, Culver City, CA, United States; ^2^The Learning Chameleon, Inc., Culver City, CA, United States; ^3^JUMP Simulation and Education Center, The Order of Saint Francis Hospital, Peoria, IL, United States

**Keywords:** teamwork, healthcare, electroencephalography, team neurodynamics, information, operating room, intubation

## Abstract

The initial models of team and team member dynamics using biometric data in healthcare will likely come from simulations. But how confident are we that the simulation-derived high-resolution dynamics will reflect those of teams working with live patients? We have developed neurodynamic models of a neurosurgery team while they performed a peroneal nerve decompression surgery on a patient to approach this question. The models were constructed from EEG-derived measures that provided second-by-second estimates of the neurodynamic responses of the team and team members to task uncertainty. The anesthesiologist and two neurosurgeons developed peaks, often coordinated, of elevated neurodynamic organization during the patient preparation and surgery which were similar to those seen during simulation training, and which occurred near important episodes of the patient preparation and surgery. As the analyses moved down the neurodynamic hierarchy, and the simulation and live patient neurodynamics occurring during the intubation procedure were compared at progressively smaller time scales, differences emerged across scalp locations and EEG frequencies. The most significant was the pronounced suppression of gamma rhythms detected by the frontal scalp sensors during the live patient intubation which was absent in simulation trials of the intubation procedure. These results indicate that while profiles of the second-by-second neurodynamics of teams were similar in both the simulation and live patient environments, a deeper analysis revealed differences in the EEG frequencies and scalp locations of the signals responsible for those team dynamics. As measures of individual and team performance become more micro-scale and dynamic, and simulations become extended into virtual environments, these results argue for the need for parallel studies in live environments to validate the dynamics of cognition being observed.

## Introduction

A shift is underway in the ways that we study the function and evolution of teams. It is being driven by the generation of multimodal biometric dynamic data streams with seconds’ resolutions, and it is expected that analyses of these data will shape our ideas about how teams are assembled, trained, and supported. (Guastello et al., 2006; Aebersold, 2018; Guastello and Peressini, 2018; Stevens et al., 2018b). In healthcare, the initial understandings of how patterns in dynamic biometric data sets relate to team member interactions and task events will likely come from simulation settings.

High-fidelity simulations provide opportunities for skill acquisition and maintenance, team training, as well as high-stakes testing, and are widely accepted today as an essential educational modality for healthcare professionals (Schmidt et al., 2013; Thomas et al., 2015; Staropoli et al., 2018). Simulation provides a mechanism for standardized clinical education across all learners, allowing exposure to critical events that clinicians might never encounter in their career in a live patient. Simulation also provides a mechanism for deliberate practice among learners. Rare but critical and time-pressured events can be recreated in a simulation, so that protocols can be established and communication problems can be identified and improved upon. Finally, simulation provides a safe environment where learners can come together as inter-professional teams to practice critical teamwork skills that are often overlooked in clinical teaching. These accomplishments have been achieved through continual refinements in simulation technology, performance measurement, and training protocols (Magee, 2003).

The shift toward more dynamic biometric models of teamwork provides an opportunity to expand our understanding of the spatial and temporal changes in team and team member cognition at a finer granularity than has been previously possible, and to approach questions that have previously been unapproachable. As these models will most likely be developed from simulation-derived data, it is important to learn how well metrics and models developed from simulated team training reflect those obtained in real-world operating room situations. Knowing if, and under what conditions, the cognitive responses for a task deviated between simulated and live patient tasks environments would provide ecologic validity for the biometric models being developed.

Where along the biometric time scale of team training (i.e., 10^−3^ to over 10^5^ s) (Salas et al., 2015) would differences be expected? The widespread use of simulations in healthcare would argue against major differences being seen between behavioral and biometric measures as these would have likely already been incorporated into simulation developments. Differences might be more expected during the execution of temporally extended episodes of action-control sequences like those found in established surgical procedures or anesthesia induction. Such episodes contain sub-sequences of actions but are mentally instantiated as one program unit (Cooper and Shallice, 2000).

The approach we have taken to investigate the detailed dynamics of such episodes are EEG-derived measures which are capable of resolving cognitive processes occurring at the milliseconds level using electrical oscillations from different regions on the scalp (Buzaki, 2006).

The metric developed, neurodynamic organization (*NO*), is the tendency of team members to enter into prolonged (>10s) metastable neurodynamic relationships as they experience disturbances to their rhythms, i.e., periods of heightened uncertainty. This metric is domain neutral and thought to occur when a team’s operating rhythm no longer supports the complexity of the task and the team needs to expend energy to reorganize into structures that better minimize the “surprise” or uncertainty in the environment (Stevens and Galloway, 2017). Consistent with this hypothesis, the frequency and magnitude of neurodynamic organizations were greater in novice teams compared with experienced submarine navigation teams (Stevens et al., 2017a).

Measures of *NO* are grounded in information theory and based on most biological signals having internal patterns and organizations. Symbolic transformations of discrete data can be used to detect and quantitate the fluctuating dynamics of these patterns (Stevens and Galloway, 2014, 2015, 2017), while information theory provides the methods for determining when and how information is created, stored, shared, and destroyed (Shannon, 1948, 1951; James et al., 2011).

A series of studies spanning high school teams to military and healthcare teams (Stevens and Galloway, 2014, 2015, 2017) has indicated that neurodynamic organizations are likely a fundamental property of teamwork. Using information theory metrics, it becomes possible to quantitatively deconstruct the neurodynamic organization of a team into the contributions of each team member (Stevens et al., 2018b). These features provide a quantitative platform for comparing the cognitive activities and live patient healthcare environments.

The goals of this study were to:

1. First, determine whether teams that performed a live patient operation (LPO) developed distinct peaks of neurodynamic organization similar to those we have previously observed during military and healthcare simulated tasks. We hypothesized that the anesthesiologist, primary neurosurgeon, and neurosurgery resident would develop discrete periods of elevated neurodynamic organization during the patient preparation and surgery, and that these elevations would occur near episodes of importance or uncertainty.
2. Second, identify whether there were times when the neurodynamic/cognitive features of the LPO team member performances diverged from those expected from similar events performed during simulations.

## Materials and Methods

### Ethics Statement

The study and the informed consent protocols were reviewed and approved by the Biomedical IRB, San Diego, CA (Protocol EEG01), and the Order of Saint Francis Healthcare Institutional Review Board, Peoria IL. All participating subjects gave written and informed consent to participate in the EEG data collections and have their data (including images and speech) anonymously analyzed per approved applicable protocols. To maintain confidentiality, each subject was assigned a unique number known only to the investigators of the study, and subject identities were not shared. This design complies with DHHS: protected human subject 45 CFR 46; FDA: informed consent 21 CFR 50.

### Simulations and Live Patient

The team members participating in both the simulation and surgery were experienced operating room staff at the Order of Saint Francis Hospital. It is likely some of them have worked together during their professional experiences, but no effort was made to quantify the level of interaction. The simulations performed were part of an integrated curriculum of airway management that was developed following a clinical needs assessment at the Order of Saint Francis Hospital in Peoria, IL. The induction, ventilation, and emergence from anesthesia is a complicated and uncertain process and one where differences in the cognition used between simulated and live patient ventilations would be detected if present.

While we have reported neurodynamic analyses of over a dozen healthcare team performances (Stevens et al., 2016, 2018b; Stevens and Galloway, 2017), in this paper, we highlight the dynamics of two, as the same anesthesiologist who performed the intubation during the live patient surgery performed two previous simulations with three intubation events.

The first simulation involved the preoperative ventilation by the anesthesiologist (AN), assisted by a circulating nurse (CN), and a scrub nurse (SN), where the mannequin exhibited an adverse response to a relative overdose of aerosolized lidocaine; this subsequently caused seizure and cardiac dysrhythmias. The immune hypersensitivity also caused swelling of the larynx which was experienced by the AN as a blockage during an initial intubation (INTB) attempt. When the transient seizure subsided, a second and successful INTB was performed. The total scenario time was 800 s.

The significant training event in the second simulation was a fire in the operating room, which required patient and staff evacuation. Prior to the fire event, the INTB in this simulation was uncomplicated. The total scenario time was 967 s.

The live patient operation to relieve pressure on the peroneal nerve was performed by a highly experienced neurosurgeon and a resident neurosurgeon. Succinctly, the surgery required an incision, an opening of the muscle fascia, the identification of the nerve, the removal of the pressure, and skin closure. The time from the patient entering the operating room (OR) until the completion of the surgery was 2,891 s.

### Electroencephalography

**Figure 1:**
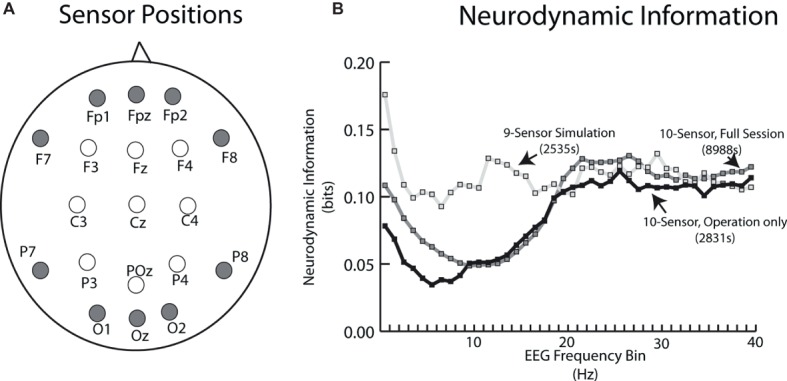
**(A)** Schematic of EEG sensor placement (looking down on the scalp) for the simulation tasks (open circles) and live patient (closed circles). **(B)** Neurodynamic information vs. EEG frequency plot for the average of the two simulation performances (open circles) and the live patient. The live patient data are plotted both for the whole task including patient removal (gray squares), as well as only during the operation (black circles).

Electroencephalography (EEG) data were collected using two EEG 10–20 systems with different sensor options (Figure 1). The 10–20 system permits uniform spacing of electrodes, independent of head circumference, in scalp regions known to correlate with specific areas of cerebral cortex. It is the standard electrode location method used to collect EEG data as well as the standard for most current databases. The simulation-derived EEG signals were acquired using a nine-sensor wet electrode system which provided coverage over the anterior, central, and posterior regions of the scalp (Figure 1A, open circles). Collecting data for the live patient procedure was constrained by the surgeon requiring a binocular loupe, and (possibly) a light source on the top of his head. Additional clearance around the ears was also needed for the stethoscope. The headband-styled 10-sensor dry electrode system used in the live patient data collection was embedded with sensors primarily in the anterior and posterior scalp regions (Figure 1A, closed circles).

A plot of the neurodynamic information at each EEG frequency bin is shown in Figure 1B. There were no significant differences in the average *NI* levels in the 18–40 Hz frequency range. The simulation sensor montage detected higher *NI* levels in the theta and alpha/mu frequency bands, due to the relative enrichment of 10-Hz team *NI* over the central scalp positions. Unless otherwise noted, subsequent comparisons between the simulation and live patient performances were made using *NI* levels from the anterior and posterior regions of the scalp and the 18–40 Hz frequency bands.

For all studies, the data acquisition began shortly after the EEG sensors were adjusted for good contact (<10 Ω). Each person’s EEG data stream were cut into segments of the simulated or live patient performance based on electronic markers inserted into the EEG data streams as well as the events observed in videos. The recorded EEG data were preprocessed using Matlab®-based FieldTrip® toolbox (Oostenveld et al., 2011), and processed as described previously (Stevens et al., 2013; Stevens et al., 2016). Signals from outside the brain can be a confounder when interpreting models built from EEG signals, especially signals obtained in complex environments. Commonly found artifacts are generated from speech, eye blinks, heartbeats, breathing rhythms, and other electromyography sources. As neurodynamic organizations regularly occur during silence, speech is an unlikely source for most organizations (Stevens and Galloway, 2014). Regular rhythms associated with eye blinks and heartbeats were identified and removed during data preprocessing (Delorme et al., 2012), and by the interactive Matlab® toolbox EEGLAB CleanLine (Mullen, 2012) plugin, which adaptively estimates and removes sinusoidal artifacts from independent components or scalp sensors using a frequency-domain (multi-taper) regression technique with a Thompson F-statistic for identifying significant sinusoidal artifacts and independent component analysis.

### Team Neurodynamic Modeling

The neurodynamic modeling is a physical to organizational – based transformation between what is observed at the team level, to the neurodynamic rhythms responsible for those behaviors. In this transformation, the physical units of EEG dynamics (i.e., microvolts) are transformed into informational units (bits) of organization. The elements of this transformation form a hierarchy that spans temporal scales from milliseconds to hours.

**Figure 2:**
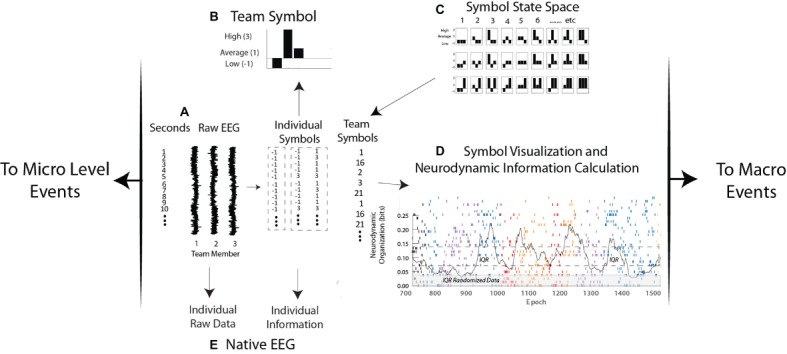
Levels of neurodynamic analyses. **(A,B)** the raw EEG signals from each person are discretized each second into low, average, and high power levels and assembled into a neurodynamic symbol. **(C)** The symbol matching the three-person power array is determined from the symbol state space lookup table and assembled into a neurodynamic data stream, where, **(D)** the team symbols are visually mapped and a moving average of entropy calculated each second. **(E)** Levels of raw EEG and normalized values (i.e., −1, 1, and 3) are calculated from the native EEG data streams.

The EEG power levels of each team member are first separated each second into high, medium, or low EEG power ranges (Figure 2A). The reporting of team member neurodynamics at a one-second resolution is in the range (250–500 ms) of functional brain connectivity associated with speech or playing guitar in duets (Stephens et al., 2010; Sanger et al., 2012), and nonverbal recognitions (Caetano et al., 2007), or approximately a half a second for a two-person action-response round trip.

For ease of visualization, the high, average, and low EEG power categories are assigned the values 3, 1, and −1. The resulting three-element array, one for each member of a three-person team, is assembled into a three-histogram neurodynamic symbol (*NS*) that represents the neurodynamic state of the team at that second. For instance, the symbol in Figure 2B indicates that at this second, team member 1 had below average, team member 2 had above average, and team member 3 had average EEG power levels. The possible combinations of three persons and three EEG power levels create a 27-symbol neurodynamic state space (*NSS*) (Stevens and Galloway, 2014; Stevens et al., 2017b). Each *NS* in the symbolic state space therefore situates the EEG power levels of each team member in the context of the levels of the other team members and the context of the task. A sequence of these symbols, the neurodynamic data streams (*NDS*) contain a neurodynamic history of the team’s performance. The granularity of the analysis can be increased by separating the EEG power into fourths or fifths with the computational costs of an exponentially increasing *NSS.*

The temporal expression of *NS* in all data streams studied has been dynamic with one subset of symbols being expressed for a minute or more, only to be replaced by another symbol subset when the task dynamics changed. These *NS* concentrations produce local variations in the randomness of the neurodynamic data streams, differences that can be quantitated by measuring the entropy over a 60-s moving window over the symbol stream that is updated each second (Figure 2D).

Entropy is the average surprise of outcomes sampled from a probability distribution or density. A *NS* density with low entropy means that, on average, the outcome is relatively predictable while a system with higher entropy would be less predictable. In this way, a dynamic and quantitative pattern of organization (in bits) can be constructed and reported with a 1-s granularity for real-time modeling, or aggregated over a performance for comparisons across teams (Stevens and Galloway, 2017).

At this point, the entropy-based units of organization have become detached from the microvolt meaning of the raw EEG signal. For instance, synchronized high-power and desynchronized low-power alpha EEG rhythms have different meanings in the context of attention and memory (Klimesch, 2012), but prolonged periods of either high or low alpha power would produce elevated neurodynamic organization and would be viewed as an organized selection of sequential actions (Cooper and Shallice, 2000).

In practice, the modeling sequence in Figure 2 first generates the three power categories for individual team members, at each sensor channel and at each of forty 1-Hz frequency bins from 1 to 40 Hz (Figure 2A). Entropy calculations across the streams of −1, 1, and 3 symbols of individual data streams produce team member neurodynamic information profiles across regions of their scalp and the EEG frequency spectrum (Figure 2E).

The scalp and frequency-wide averages of the team *NDS* initially pinpoint periods of higher neurodynamic organization which can then be linked with task events. This initial step is followed by deconstruction of the team data into each team member’s sensor and frequency dynamics around regions of interest (Stevens et al., 2018a). The total number of parallel data streams for a three-person team with every individual wearing a 10-sensor EEG headset, this would be 400 team *NDSs* and 1,200 individual team member *NDSs,* as well as a similar number of parallel entropy data streams.

As increased organization is accompanied by decreased entropy, the individual and team entropy values are subtracted from the maximum entropy for the number of symbols being modeled, i.e., 3.17 bits for 9 symbols or 4.775 bits for 27 symbols, and the resulting values are termed neurodynamic information (*NI*); this procedure makes increased neurodynamic information and increased organization both positive values.

## Results

### Team and Team Member Neurodynamics During Simulation Training

**Figure 3:**
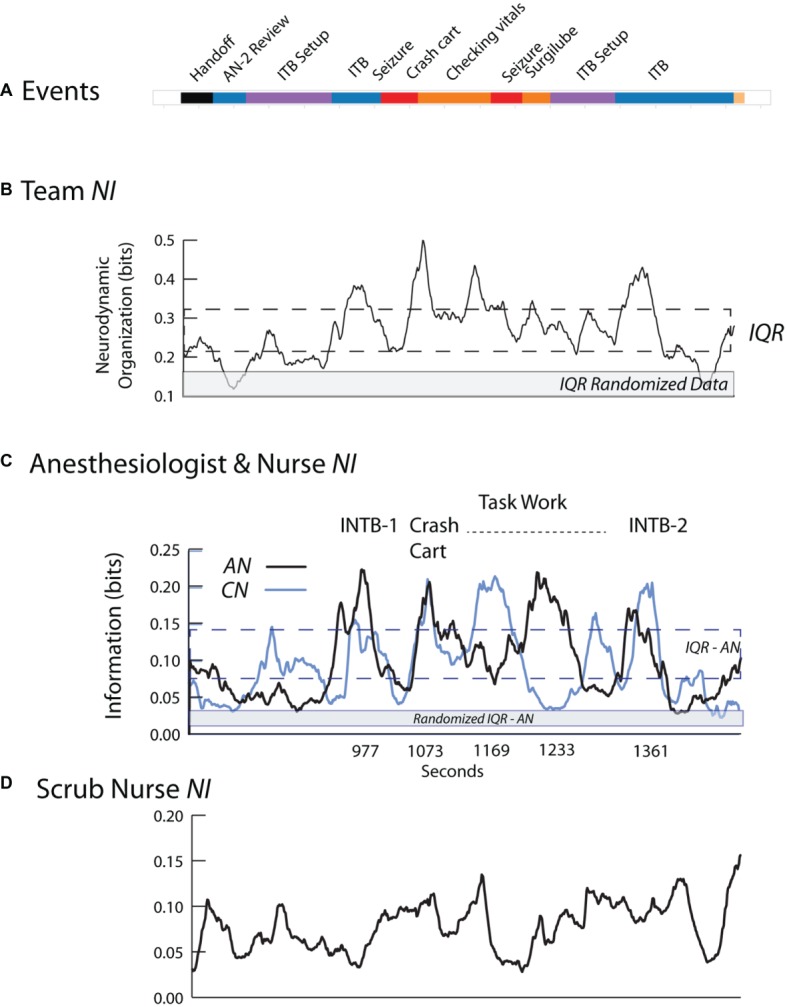
Team and individual neurodynamics during a healthcare simulation. **(A)** The task event segments. **(B)** A quantitative neurodynamic information profile is plotted for the team. The *NI* is a profile of the average bits of information using all sensors and frequencies. The dotted lines indicate the interquartile range (IQR), i.e., 25–75% of the data values, and the gray line indicates the IQR for the randomized data. **(C)** The *NI* traces of the AN (dark) and CN (light) during the simulation with selected events labeled. **(D)** The *NI* trace of the SN.

Tracing the frequency, magnitude, and duration of fluctuations in neurodynamic information provides a quantitative history of a team’s neurodynamic responses to events that triggered the team to neurodynamically reorganize. The *NI* fluctuations of an experienced anesthesiology team performing a complicated sequence of ventilation procedures during a simulation are shown in Figure 3. The events in this simulation included an early unsuccessful INTB attempt (INTB-1), patient seizures requiring a call for a Crash Cart, and a second (successful) INTB attempt (INTB-2) (Figure 3A). This example was chosen from others available (Stevens et al., 2016) as the AN performing this simulation had performed a similar procedure during a second simulation, and was also responsible for intubating the patient during the surgery.

The team *NI* neurodynamic profile was low until 920 s and then increased during the first intubation attempt (Figure 3B). After decreasing over the next 100 s, the *NI* again increased in response to the patient seizing, and remained near the top of the interquartile range (IQR) and then decreased before peaking again during the second intubation attempt.

The heterogeneity underlying the team neurodynamic profile was shown by deconstructing the team *NI* into that of each team member using information theory approaches (Stevens et al., 2018b). There were three *NI* peaks where the AN and CN showed coordinated NI dynamics and these were the first intubation attempt (*r* = 0.75 with AN leading CN at 30 s), the episode of seizure (*r* = 0.84) and the second intubation (*r* = 0.70 with AN leading CN at 10 s). This coordinated behavior decreased during the middle of the task, i.e., between the seizure episodes and the second intubation. The *NI* of the SN (Figure 3D) showed few defined fluctuations in response to the evolving task, and also little coordination with the dynamics of AN or CN.

For each primary event, the AN made comments indicating uncertainty including:

1. *INTB-1*: “There is pus or something in the trachea or an obstruction, I can’t tell which; I think I am going to have to go through it, do it with the trachea tube… It looks like he is seizing.”
2. *Seizure V-tach*: “Ok, that’s not unexpected. Let’s go ahead and take this out if he is going into tach.”
3. *Seizure/INTB-2*: “I am not sure what my other options are. Because he has a history of seizures I think we are out of drugs.”
4. *INTB-2*: “There is something in the trachea… I am not sure if I can see if it is a foreign body or…”

These results suggest that events likely to increase team or individual uncertainty are also those that raise *NI* levels; in other words, *NI* may act as a barometer for the uncertainty for each member, and by extension, for the team (Stevens et al., 2016).

The coordinated neurodynamics between the AN and CN during events requiring cooperation, yet independent neurodynamics while performing individual tasks, also suggest the possibility of being able to separately identify periods of teamwork and taskwork. Lastly, simulation-based neurodynamics may help refine what meaningful information for a team member might be. While the SN was watching, and likely understood the details of the different task episodes being performed, without her actual involvement, both the neurodynamic coordination with the AN and CN and the peaks of elevated *NI* were missing. That is, the task events that will increase *NI* have to be meaningful for a person, not just interesting.

### Team Neurodynamics During a Live Patient Surgery

The surgical team in this example consisted of the AN who had previously performed ventilation procedures during simulation training, an experienced neurosurgeon (NS1) and a neurosurgery resident (NS2), a surgical nurse (SN) and a circulating nurse (CN); EEG data were collected and modeled for the AN, NS1, and NS2 for this example.

**Figure 4:**
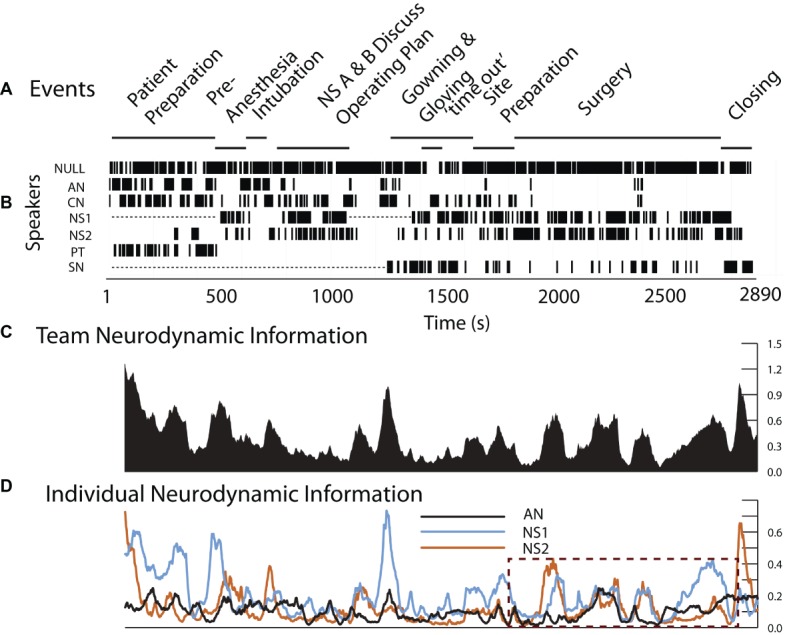
Team and individual neurodynamics during a peroneal nerve decompression surgery. **(A)**. Task events. **(B)**. Team member speech. **(C)**. The team *NI* profile using the average bits of information from all sensors and frequencies. **(D)**. The *NI* traces of the AN, NS1, and NS2. The dotted rectangle indicates the period of the surgery.

As shown in Figure 4, the operating room setting differed from most simulations by lasting three times longer than simulations like that in Figure 3. There were also prolonged periods when team members were outside the room as indicated by the dotted lines in the Speakers row (Figure 4B). This did not affect EEG collection which was being recorded on a headset chip, but it interfered with the ability to link the EEG with events during those periods.

If the observed simulation neurodynamics were accurate representations of those occurring during surgery, then with the operating room team, we would expect to see:

1. The presence of discrete *NI* peaks near important events.
2. The differential responses of team members to these events.
3. Aligned team member *NI* fluctuations during coordinated activities.

**Figure 5:**
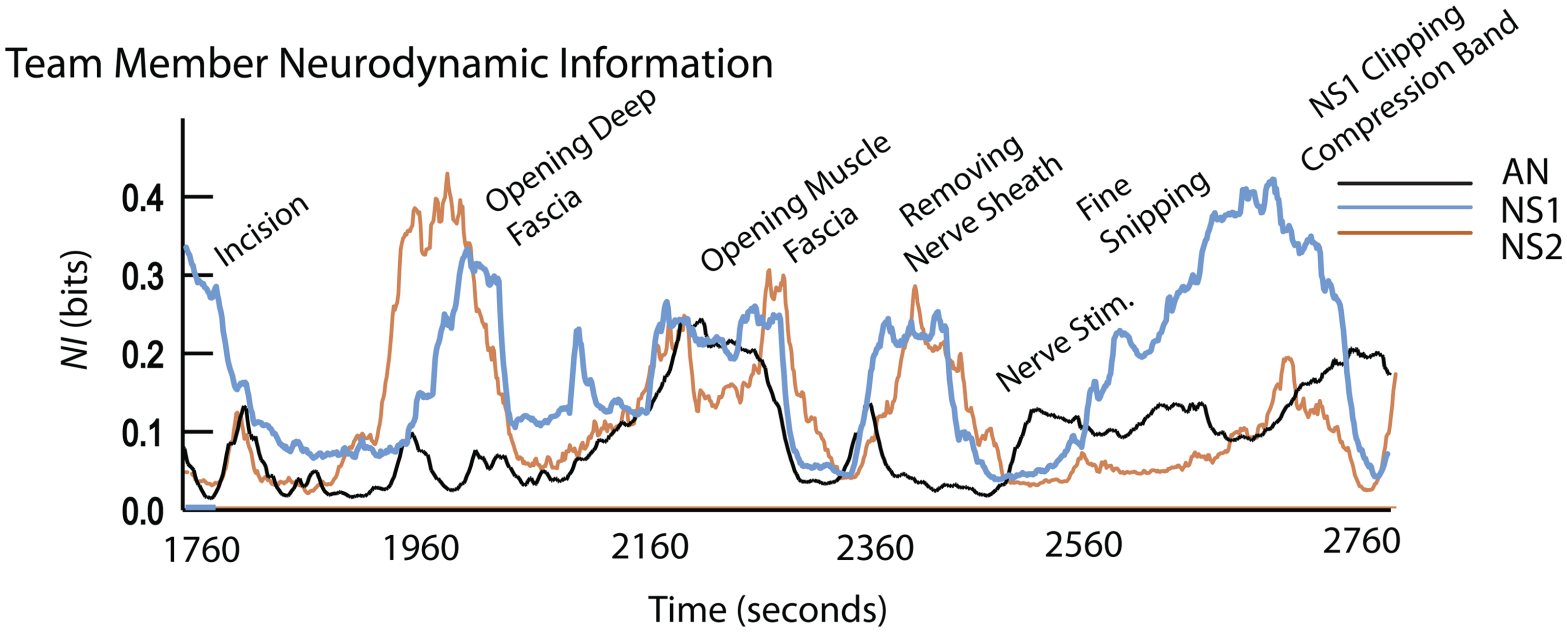
Event details and team member *NI* profiles during surgery.

Consistent with the first goal, the neurodynamics of the surgery team showed discrete peaks of increased *NI* during the preoperative patient ventilation as well as surgical preparation and subsequently during the surgery (Figure 4C). The deconstruction of the team *NI* into those of the AN, NS1, and NS2 showed periods of individual and coordinated *NI* dynamics, especially during the surgery as shown in the dashed outline (Figure 4D). These are investigated further in Figure 5.

The surgical sequence for a peroneal nerve decompression begins with an incision, the spreading of the incision, and the opening of the underlying fascia. The nerve is then identified, isolated, and stimulated if necessary. The tissue source of the compression is then identified and removed.

The early surgical segments (until ~2,500 s) were performed by NS2 assisted by NS1. During the surgery, there were three episodes of correlated *NI* between NS1 and NS2 (*r* = 0.79 at a 20-s cross-correlation lag around 1980s), *r* = 0.43 at ~2,300 s, and *r* = 0.75 at ~2,400 s), and these occurred while the neurosurgeons worked closely together. After the nerve was isolated and the source of the nerve compression was identified, NS1 performed the removal of the compressive block (from 2,460 to 2,709 s); during this final procedure, only the *NI* of NS1 was elevated.

The neurodynamic similarities in the *NI* profiles derived from the simulation and live patient-derived conditions indicate that at the level of temporal dynamics, the simulation-acquired data provide an accurate representation of the types of neurodynamics that will be observed in real-world situations. The coordinated *NI* dynamics between NS1 and NS2 are similar to those seen between the AN and CN in Figure 3, therefore substantiating simulations cognitive - ability to evoke neurodynamic correlates of teamwork.

The next analysis examined the degree of neurodynamic heterogeneity present in the extended period of *NI* associated with the removal of the source of nerve compression. The analysis during this 4-min period searched for across-frequencies temporal changes as well as across-the-scalp spatial changes in *NI* dynamics.

**Figure 6:**
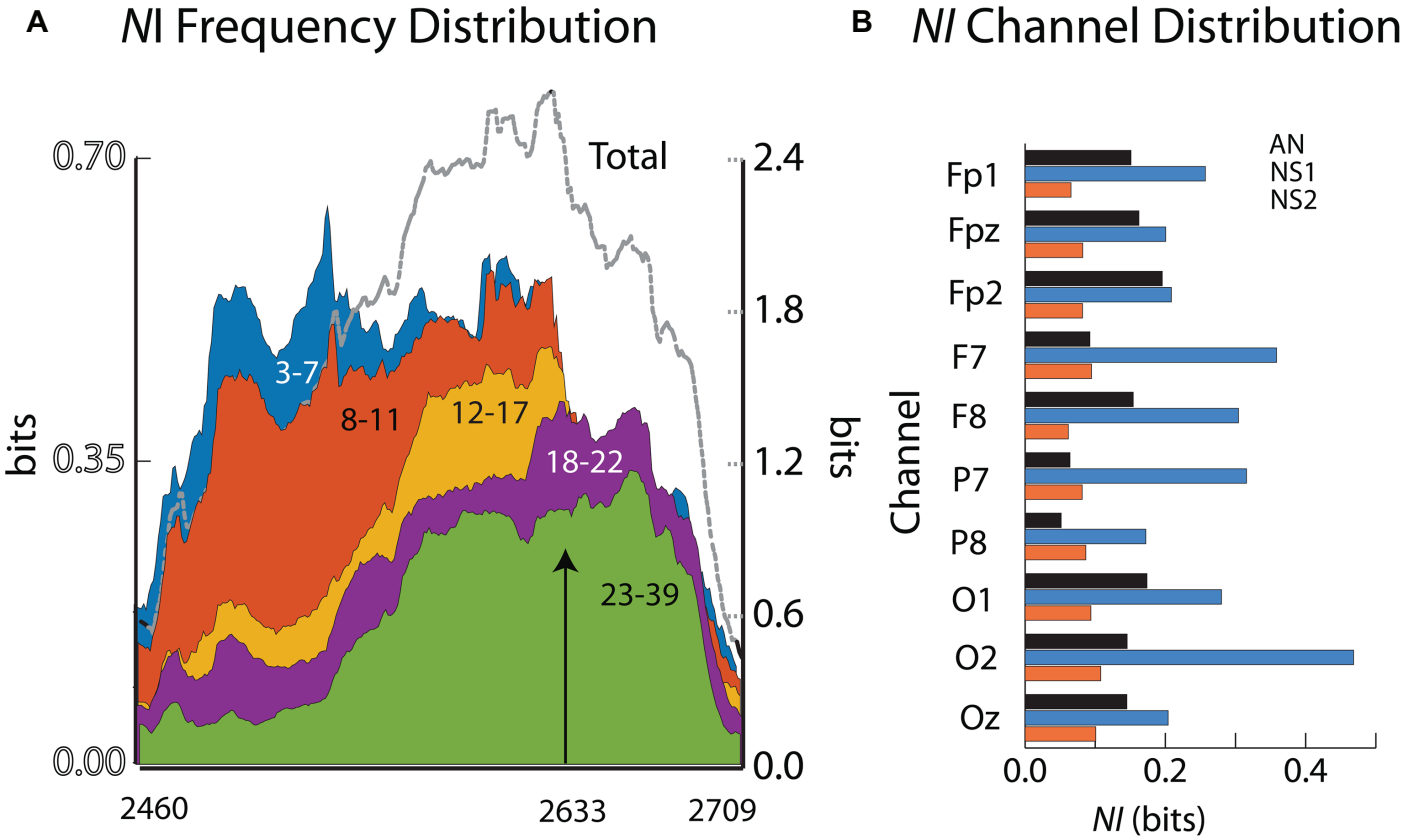
**(A)** The *NI* values for the different frequency ranges are plotted for the final surgical procedure (2460–2,709 s). The arrow indicates when the surgeon completed his operation. **(B)** The across-frequency and sensor *NI* averages for the 10 EEG sensors. The member order in each bar cluster is AN, NS1, NS2.

The aim of these analyses was to determine if there was a neurodynamic trajectory from the initiation of the procedure, through the peak period of neurodynamic information, to the return to a neurodynamic baseline. Neurodynamic information profiles were generated for five EEG frequency bands: delta/theta (3–7 Hz), alpha (8–11 Hz), mu (12–17 Hz), low beta (18–22 Hz), and high beta/gamma (23–40 Hz). The earliest and largest *NI* levels were in the 3–7 Hz (delta/theta) and 8–11 Hz (alpha) frequency bands and these remained high until 2,633 s when they abruptly declined (Figure 6A). Coincident with this decrease was NS1 completing the removal of the compressive block on the nerve. The beta and gamma frequency bands predominated after this period and then declined to baseline levels over the next minute.

**Figure 7:**
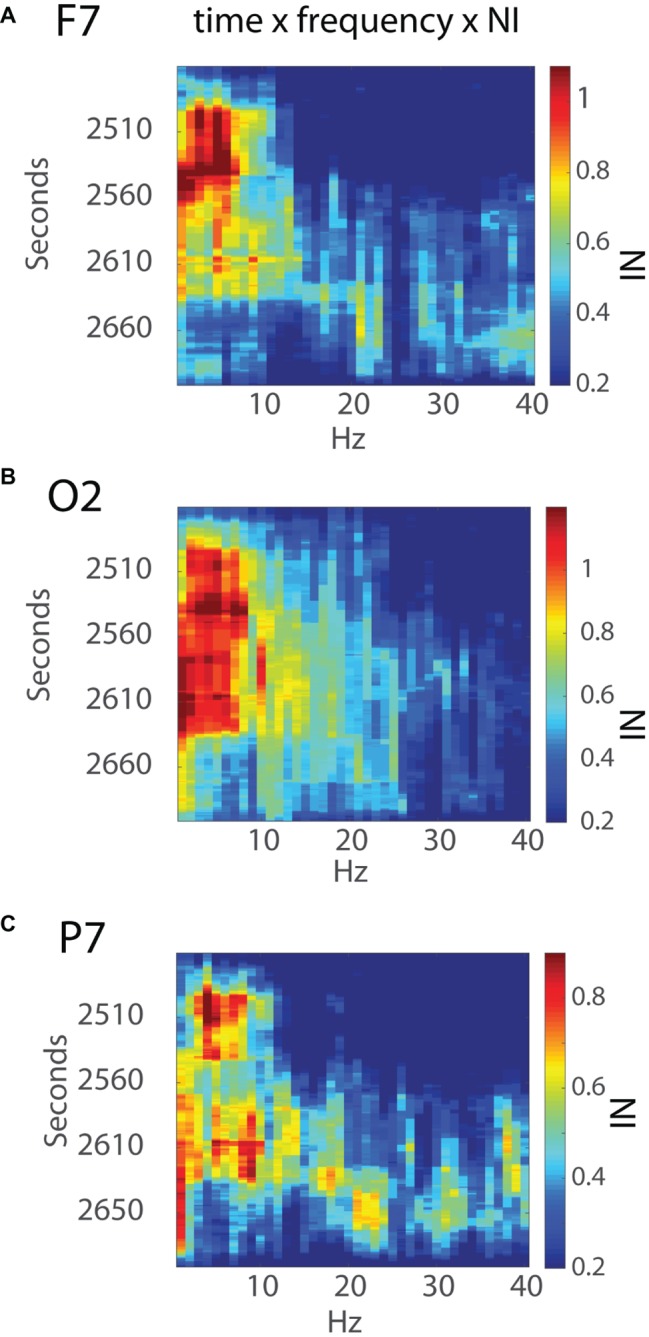
Time x frequency vs. *NI* levels for the **(A)** F7, **(B)** O2, and **(C)** P7 sensors. The NI levels are shown by the color bars to the right.

The *NI* levels during these 4 min were greatest at sensors O2, F7, P7, and F8 (Figure 6B). The analyses were refined by generating time x frequency x *NI* plots for the F7, O2, and P7 sensors to explore the temporal and spatial sequencing of *NI* levels across sensors and frequencies (Figure 7).

Early *NI* increases were detected at the F7, P7, and O2 sensors ~30s into the final surgical procedure and were mostly in the 3–11 Hz range. The *NI* levels at the P7 sensor were short lived and followed by *NI* decreases at the F7 sensor. In contrast, the O2 *NI* levels continued to increase during the next 2 min and extended toward higher frequencies. At epoch 2,633 s, the 3–11 Hz *NI* abruptly stopped at the O2 sensor, which, as described earlier, occurred after the alleviation of the nerve compression. During the remaining time before closing the incision, there was an *NI* increase in beta and gamma frequency bands, particularly at the P7 sensor.

### Neurodynamics of the Anesthesiologist During the Intubation Events

The analyses of the peroneal nerve decompression surgery in Figures 6, 7 illustrate the neurodynamic heterogeneity within an extended period of uncertainty, and show how this heterogeneity can be used to describe the surgical procedure in terms of a spatial and temporal neurodynamic trajectory. To explore the generality of these findings, a similar analysis was performed upon another critical event during the operation which was the patient intubation procedure. The anesthesiologist who performed the patient intubation during the operation previously performed three intubations under simulated conditions while acquiring EEG data that allowed neurodynamic comparisons across training modalities.

**Figure 8:**
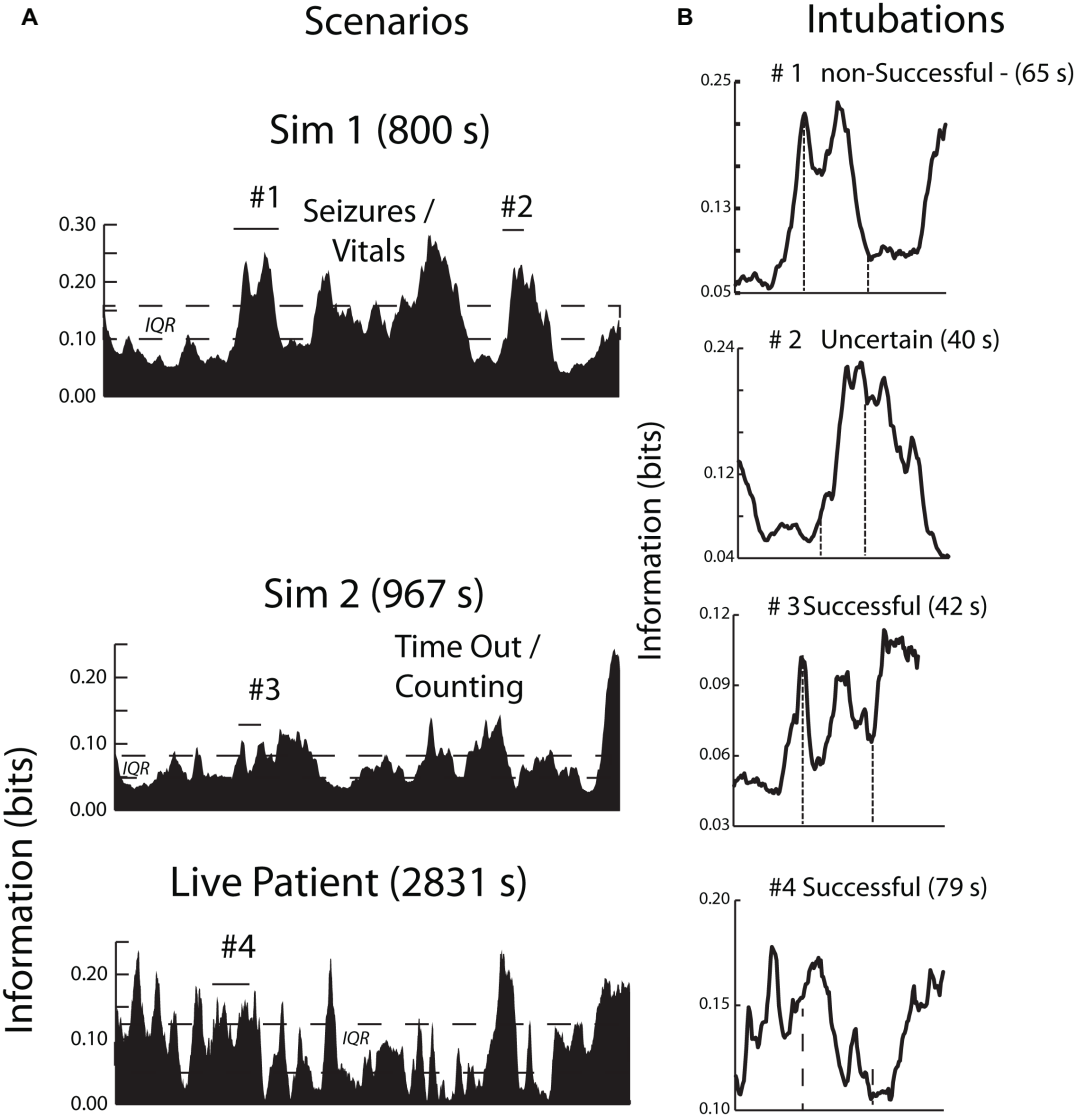
**(A)** The contexts of the INTB activities are shown by the area plots of the scenario *NI*; the periods of intubation are shown by horizontal lines. **(B)** This figure compares the neurodynamic information profiles of three simulated INTB attempts of varying difficulty with a live patient INTB attempt.

The simulated and the live patient INTB segments were identified and isolated after bracketing them within 60-s data sections before and after the procedure to provide a dynamic context. Each of the INTB segments were above the IQR range for the performance indicating the procedure was one of importance for the anesthesiologist during both the simulations and in the operating room (Figure 8A). The four INTB segments ranged from 40 to 79 s in length and within each of the segments, there were peaks in the *NI*, often biphasic. One of the intubations (#1 of Figure 8B) was unsuccessful due to a blockage and the second intubation (#2) could not be confirmed as successful before the simulation ended. The other simulated and live patient intubations were successful. Aside from the elevated *NI* levels, there were no consistent defining features of the INTB procedures, which was not surprising with the temporal and intubation outcome differences among the trials.

**Figure 9:**
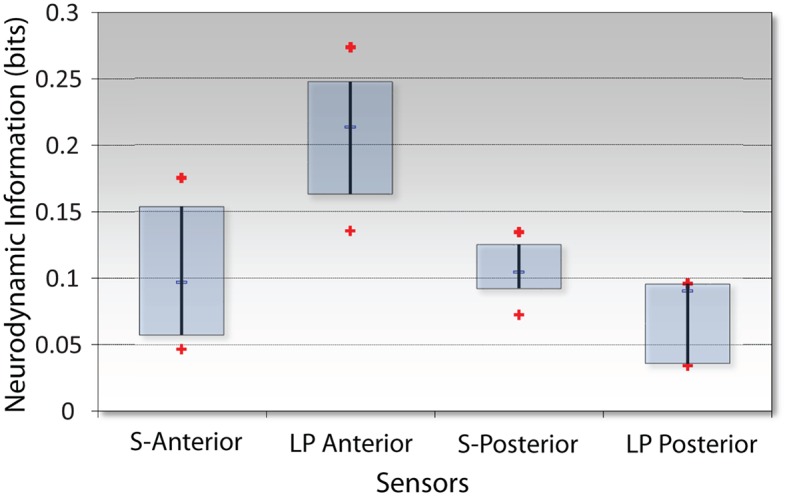
*NI* levels at the anterior vs. posterior channels for the simulation (S) or the live patient (LP) INTB procedures. The frequency-averaged (18–40 Hz) *NI* levels were measured at the anterior (F3, Fz, F4) or posterior (P3, Pz, and P4) sensors for the simulation tasks, and the anterior (Fp1, Fpz, Fp2, F7, and F8) or posterior (P7, O1, Oz, O2, and P8) sensors for the live patient INTB.

The analytic focus next shifted to the sensor *NI* levels during the INTB events. Because of the differences in the simulation and LPO EEG montages (Figure 1), these analyses contrasted the *NI* levels of the anterior and posterior sensors. These analyses were performed using the data from the INTB windows shown in Figure 8B. The anterior vs. posterior sensor regions’ *NI* levels for the simulation INTB events were not significantly different (*Z* = 0.77, *p* = 0.44, Wilcoxon), while the *NI* levels for the live patient INTB were nearly 3-fold greater at the anterior than posterior regions (*Z* = 2.02, *p* < 0.05) (Figure 9). The anterior sensor *NI* levels were also significantly greater than the simulation groupings, indicating a skewing of the brain-wide neurodynamic organization toward the anterior regions during the live patient INTB procedure.

**Figure 10:**
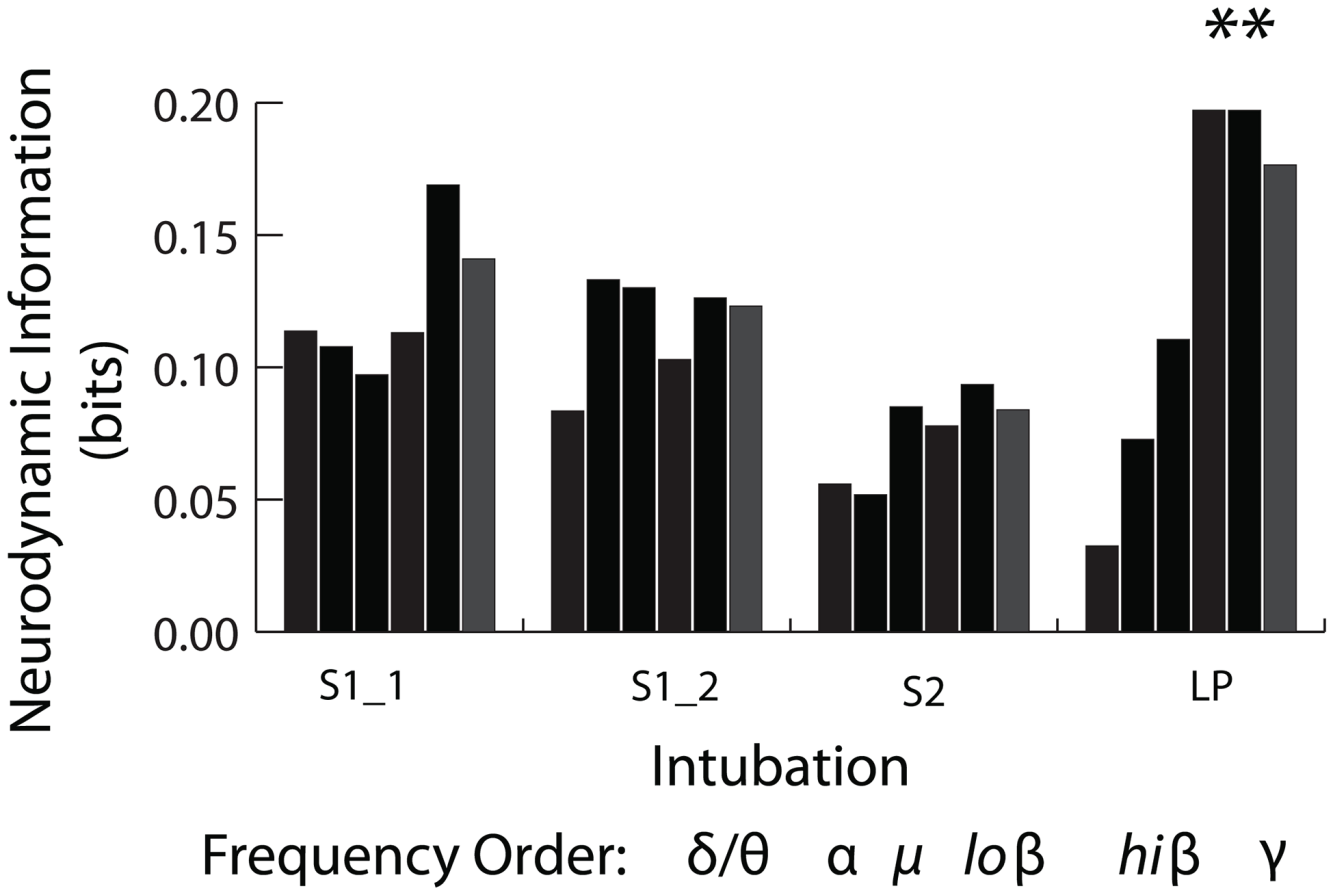
Frequency band distribution of *NI* for the INTB events. The pooled low beta, high beta and gamma frequency bin *NI* levels from the live patient INTB were significantly greater than those from the simulations (Mann Whitney*, Z =* 2.4*, p =* 0.01).

The frequency band *NI* distributions were next generated across the 1–40 Hz spectrum shown in Figure 10. The *NI* values were binned into the delta/theta (3–7 Hz), alpha (8–11 Hz), mu (12–17 Hz), low beta (18–22 Hz) high beta (23–32 Hz), and gamma (33–40 Hz) bins. These comparisons were made using only the data from the INTB windows shown in Figure 8.

As previously described, *NI* is a measure of the organizational patterns in a neurodynamic data stream. As such, they could represent persistent patterns of elevated, depressed, or intermediate EEG power levels by a team member or a team. Making this distinction is important as elevated gamma power has been associated with memory retrieval (Vergauwe and Cowan, 2014), whereas gamma power suppression has been associated with focused attention and while reading for comprehension (Lachaux et al., 2008; Ossandon et al., 2011; Sato and Mizuhara, 2018).

**Figure 11:**
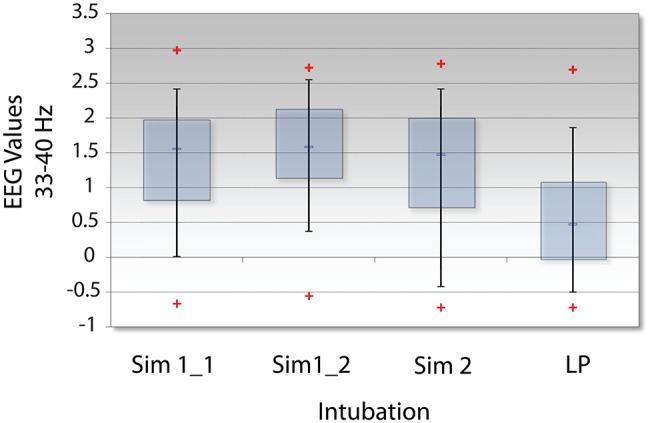
Levels of EEG-gamma power during INTB events. The raw EEG values were determined for each of the intubation events; LP = live patient.

Analyses were therefore performed using the high, average, or low EEG values (i.e., −1, 1, or 3) rather than *NI* levels. Figure 11 indicates that the elevated EEG beta-gamma *NI* levels found during the live patient INTB were due to low gamma EEG power values (*H* = 137, df = 3, *p* < 0.01) compared with the above average gamma power values during the simulation.

## Discussion

The results indicate that the sensor and frequency-averaged profiles of team and team member neurodynamics were similar in both the simulation and live patient environments. This provides an important validation of previous studies with military and healthcare teams where the team neurodynamics were linked with speech (Gorman et al., 2016), stressful situations (Stevens et al., 2013), and expert performance ratings (Stevens and Galloway, 2017) during high-fidelity simulation training. They further suggest that developing models to track the appearance of these fluctuations or estimate/predict their magnitude and duration could have practical training applications. For instance, providing these neurodynamic profiles to instructors prior to a debriefing following a training exercise could help focus the discussions around periods where the team might have experienced uncertainty. Similarly, the periods of elevated *NI* could serve as triggers for providing feedback in an intelligent tutoring setting for optimizing team health and performance.

While the overall neurodynamic profiles were similar under simulated and live patient conditions, according to the ideas behind hierarchal cognition, each *NI* peak is likely neurodynamically heterogeneous. The appearance of patterns of elevated *NI* with the onset of meaningful events and their decline after the task completion are consistent with the idea they are neurodynamic representations of a set of procedures or subtasks needed to complete a task, i.e., a mental episode. Mental episodes are typically extended periods, with a defined beginning and ending, of focused deliberate behavior during which a sequence of steps are completed (Schneider and Logan, 2015). The execution of episodes is thought to begin by loading a sequence representation of the task into memory, which controls the sequence and identify of the subtasks. Following the ideas of hierarchical cognition, the component sequences are then executed (Schneider and Logan, 2006).

An example of this heterogeneity, and the episodic nature of the final surgical procedure, is shown in Figures 7, 8 where the neurodynamics revealed a change in the neurosurgeons cognitive state with the onset of the final surgical procedure. The primary focus for this neurodynamic reorganization was the occipital lobe at the 3–11 Hz frequencies. A second major cognitive state change occurred when the surgery was completed and the occipital lobe neurological organizations were replaced by a more heterogeneous frequency profile at the P7 channel before returning to preoperation levels. A similar neurodynamic analysis of the intubation procedure performed by the anesthesiologist suggests that each *NI* peak might show neurodynamic complexity at the sensor and frequency level.

The *NI* levels during the live patient INTB were unequally distributed between the anterior sensors where the levels were significantly greater than those from the posterior sensors. The anterior and posterior sensors’ *NI* levels from simulation attempts were not statistically different, but were intermediate to those at the anterior and posterior levels during the surgery.

The finding of elevated neurodynamic organization in the frontal regions during INTB may be significant as frontal regions have been implicated in the detection of unfavorable outcomes, error correction, and resolution of uncertainty, all of which might be expected to play a role during this critical procedure (Ridderinkhof et al., 2004; Murray and Rudebeck, 2017). The EEG frequencies associated with the elevated frontal sensor *NI* were in the low beta – low gamma frequency range. Gamma EEG rhythms, or “gamma oscillations” emerge from neuronal structures at rates from 30 to up to 300 Hz. Their rhythms are driven by balances of inhibitory GABAergic interneurons and excitatory glutamatergic neurons (Whittington et al., 1995). Gamma oscillations occur alongside and in proportion to perceptual processes/salience (Sedley and Cunningham, 2013) and are thought to be pivotal in: (1) the search for information, or the refreshing of information within the brain, and (2) the communication of this information across regions of the brain.

The suggestion of gamma rhythm involvement in the search for information to populate short-term memory is based on repeated observations showing decreased response speed with the number of items in short-term memory, reaching a processing rate limit of 25–30 items per second (Vergauwe and Cowan, 2014). These authors have proposed that information for features of one item are represented by groups of neurons that fire within a gamma cycle and this gamma-band synchronization facilitates neural communication and synaptic plasticity.

Gamma rhythms do not act in isolation during this neural communication, but become phase locked and nested within theta rhythms (~ 5–7 gamma per theta wave) or alpha oscillations which serve to segment neuronal representations in time, and perhaps support their coordinated action across neuronal assemblies (Bonnefond and Jensen, 2015). In these two instances, gamma activity increases.

It is also becoming clear that attention-demanding tasks like reading for comprehension not only activate specific cortical regions, but also deactivate others that might interfere with the task either at local (Klimesch, 2012) or more distant cortical regions (Farooqi and Manly, 2018). Studies using intracerebral electrodes have suggested that focused interaction with the external world is associated with gamma rhythm suppression in the default mode network (Ossandon et al., 2011). This is a series of brain regions linked with introspective thoughts (Raichle et al., 2001).

Possible linkages between the reduced gamma rhythm levels we have observed during the INTB event of the live patient and previously reported spatially localized network and short-lived gamma suppression are difficult to speculate on from a single sample. The possibility exists however that the INTB with the live patient induced a more attentive state in the AN than that provided by the simulations, suggesting a fundamental difference in the two environments.

As expressed by the AN: “I was aware that the OR was a real patient and the lab case was just a simulation. I felt the usual urgency in the real case to perform well as opposed to the lab simulation where it’s more relaxed because you know there isn’t anything important at stake.” As measures of individual and team performance become more micro-scale and dynamic, and simulations become extended into virtual environments, these results argue for the (at least limited) need for parallel studies in live environments to maximize the benefits from these emerging technologies.

## Ethics Statement

The study and the informed consent protocols were reviewed and approved by the Biomedical IRB, San Diego, CA (Protocol EEG01), and the Order of Saint Francis Healthcare Institutional Review Board, Peoria IL. All participating subjects gave written and informed consent to participate in the EEG data collections and have their data (including images and speech) anonymously analyzed per approved applicable protocols. To maintain confidentiality, each subject was assigned a unique number known only to the investigators of the study, and subject identities were not shared. This design complies with DHHS: protected human subject 45 CFR 46; FDA: informed consent 21 CFR 50.

## Author Contributions

RS and TG acquired and processed the EEG data for the simulation and live patient performances then performed the neurodyamic modeling and generated and conducted the data analysis. AW-D oversaw the development and implementation of the team simulation activities. All authors participated in preparing the paper.

### Conflict of Interest Statement

The authors declare that the research was conducted in the absence of any commercial or financial relationships that could be construed as a potential conflict of interest.
